# Dynamic Heterogeneous User Generated Contents-Driven Relation Assessment via Graph Representation Learning

**DOI:** 10.3390/s22041402

**Published:** 2022-02-11

**Authors:** Ru Huang, Zijian Chen, Jianhua He, Xiaoli Chu

**Affiliations:** 1 School of Information Science & Engineering, East China University of Science and Technology, Shanghai 200237, China; zj.chen@mail.ecust.edu.cn; 2 School of Computer Science and Electronic Engineering, University of Essex, Colchester CO4 3SQ, UK; j.he@essex.ac.uk; 3 Department of Electronic and Electrical Engineering, University of Sheffield, Sheffield S10 3JD, UK; x.chu@sheffield.ac.uk

**Keywords:** user-generated contents, relation assessment, community detection, graph representation learning

## Abstract

Cross-domain decision-making systems are suffering a huge challenge with the rapidly emerging uneven quality of user-generated data, which poses a heavy responsibility to online platforms. Current content analysis methods primarily concentrate on non-textual contents, such as images and videos themselves, while ignoring the interrelationship between each user post’s contents. In this paper, we propose a novel framework named community-aware dynamic heterogeneous graph embedding (CDHNE) for relationship assessment, capable of mining heterogeneous information, latent community structure and dynamic characteristics from user-generated contents (UGC), which aims to solve complex non-euclidean structured problems. Specifically, we introduce the Markov-chain-based metapath to extract heterogeneous contents and semantics in UGC. A edge-centric attention mechanism is elaborated for localized feature aggregation. Thereafter, we obtain the node representations from micro perspective and apply it to the discovery of global structure by a clustering technique. In order to uncover the temporal evolutionary patterns, we devise an encoder–decoder structure, containing multiple recurrent memory units, which helps to capture the dynamics for relation assessment efficiently and effectively. Extensive experiments on four real-world datasets are conducted in this work, which demonstrate that CDHNE outperforms other baselines due to the comprehensive node representation, while also exhibiting the superiority of CDHNE in relation assessment. The proposed model is presented as a method of breaking down the barriers between traditional UGC analysis and their abstract network analysis.

## 1. Introduction

Nowadays, user-generated contents (UGCs) are riddled in various large-scale online platforms such as e-commerce platforms, discussion forums, live streaming platforms and social networks [1,2,3,4]. The research on UGC can be roughly divided into intrinsic quality improvement and their interrelation analysis, which are indispensable parts of the online decision-making platform. Normally, researchers focus on the possible contents distortion or quality degradation, while neglecting the importance of relation assessment among various UGCs. Tapping into relationships of those high-quality UGCs can attract general attentions and produce great social benefits. In reality, many tangled contents can be described and analyzed by the relevant characteristics of a complex network.

The research on entities’ relationships via abstract network structures has always been a hotspot in many fields [5,6,7,8]. Accurate relation assessment and prediction are helpful to analyze the UGC network evolution patterns and assist network maintenance, which is of great significance to enhance the survivability and to improve the reliability in both static and dynamic networks. More precisely, relation prediction in network refers to forecasting the underlying existence of a link between two nodes based on the network structural information and the intrinsic information of nodes [9].

Among the existing relation prediction methods, the heuristic methods measure the connectivity of two nodes and the graph statistical features from the perspective of similarity, such as the degree of node, node centrality and the clustering coefficient [10,11], while the graph representation learning methods concentrate on encoding the intricate network structures into low-dimensional vector space so as to capture the multi-scale and high-level node features from the underlying topology [12]. These approaches [13,14,15], on the other hand, mainly represent the network under the assumption that the network is homogeneous, while focusing on the static attributes of the network. Actually, the abstract UGC network in the real world is normally composed of multiple types of nodes or edges, while the relationship between nodes is complex and evolves over time, which presents heterogeneous and dynamic characteristics. This renders the straightforward application of most existing relation prediction methods infeasible [16].

**Figure 1:**
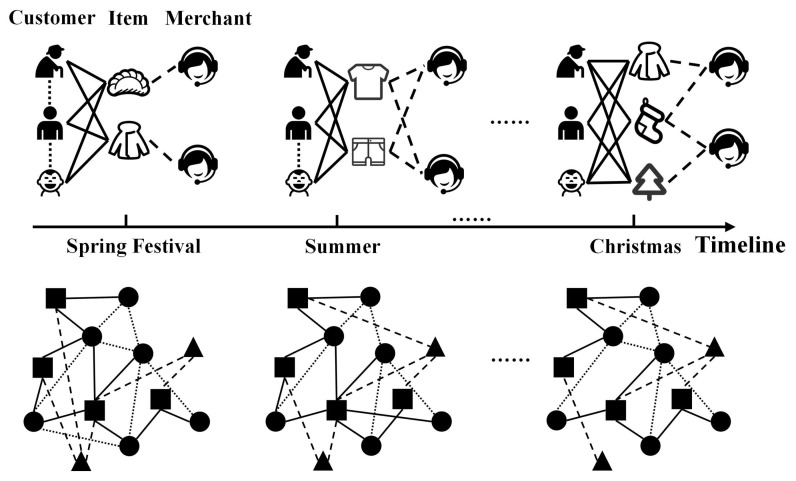
A user generated dynamic heterogeneous network example: a commodity supply–demand network and its generalization of abstract graph structure. The solid line denotes the relations between customers and items, the dotted lines indicate the relations between customers, and the dashed lines indicate the relations between merchants and the produced items.

Dynamic heterogeneous networks are composed of different types of entities and relations, which usually evolve over time. Taking Figure 1 as an example, above the timeline is a commodity supply–demand network containing three types of nodes (i.e., customer, item and merchant) and edges (i.e., customer–customer, customer–item and merchant–item). The demands for T-shirts and shorts are greater in summer, while, as Christmas approaches, customers show demands for other products, such as Christmas trees and stockings, which demonstrates how the structure of networks varies over time with dynamic characteristics.

Up to now, limited attempts have been made to investigate the embedding of dynamic heterogeneous networks. Kong et al. [17] used the graph convolutional network (GCN) to extract the spatial structural features from heterogeneous information networks (HINs) and employed the long short-term memory (LSTM) network to forecast the existence of links. However, this shallow GCN merely captured neighbors with low proximity while ignoring the heterogeneous characteristics, and cannot be stacked for multiple layers due to the over-smoothing problem. DHNE [18] devises a historical–current networks structure, which takes all neighbors in the time step into consideration in order to learn the latent node embedding from the dynamic condition; moreover, a metapath-based random walk is conducted to capture the heterogeneous semantic information. DyHATR [19] integrates a hierarchical attention module and recurrent neural network based model to learn heterogeneity as well as temporal evolution. More recently, Ji et al. [20] introduce a Hawkes-process-based method to model the formation process of heterogeneous events adequately and use the importance sampling strategy to capture representative events for influence propagation. Xie et al. [21] propose the DyHINE method, comprising a temporal dynamic embedding module and an online updating module, which can deploy real-time updated embedding when the network evolves.

Although the above approaches work well in many types of applications, they still have some drawbacks. In general, traditional matrix factorization methods usually perform relatively poorly due to the high computational cost of decomposing the massive matrix, while most graph embedding techniques mainly focus on the homogeneous network. To address this challenge, our dynamic heterogeneous graph embedding method tends to learn a map function that converts complicated input networks into low-dimensional space for better representation while capturing the evolutionary properties of networks. The Markov-chain-optimized metapath is able to preserve the heterogeneous structure and semantics while improving computational efficiency. Despite multi-scale features on networks have been thoroughly explored, the current network embedding methods solely consider the low- or high-order proximity characteristics of nodes in a limited perspective, while ignoring the global features represented by community structures. To solve the structural information loss in feature extraction, we not only integrate other side information, but also capture global structure semantics via a clustering technique. Moreover, there is a lack of research about capturing temporal evolution characteristics, which is also of great importance in dynamic relation prediction. To address this issue, we propose an encoder–decoder structure to learn the temporal dependencies after obtaining the nodes representation, which contains comprehensive heterogeneous topology information and can be delivered for distilling the implicit correlations between each time step. Our main contributions can be summarized as follows:

- **User generated contents-driven method:** We proposed a graph representation learning-based method, named the community-aware dynamic heterogeneous graph embedding method (CDHNE), for predicting and assessing the relationships between different user-generated contents. We consider the generalized user-generated contents as abstract nodes, which form a dynamic heterogeneous network, so as to introduce the graph embedding methods. The objective of this work is to explore the semantics of human activities and perform a comprehensive relation assessment of these activities.
- **Multi-level representation learning:** We facilitated the metapath-based random walk, utilizing Markov chain approximation, for localized heterogeneous contents learning. An edge-centric attention mechanism is introduced for subgraph-level feature aggregation. The clustering technique, depending on node embedding, offers effective global structure semantic extraction without prior information.
- **Temporal dynamics extraction:** We devised an encoder–decoder structure with two variants, i.e., CDHNE-GRU and CDHNE-LSTM, to learn the temporal evolutionary patterns in dynamic heterogeneous networks. Concretely, we split the dynamic heterogeneous network into several snapshots and leveraged the recurrent memory unit to capture long-term dependencies over time steps. In each hidden unit, the generated parameters are delivered to trigger the next gate. Finally, we can obtain the output through a fully connected decoder.
- **Experimental results:** We constructed datasets containing a series of human activities, which included academic collaboration, commercial promotions and social interactions, and conducted extensive experiments to demonstrate the effectiveness of CDHNE under the user-generated contents scenarios. Specifically, we evaluated our proposed model on relation prediction problems and conduct community detection tasks to validate the effectiveness of CDHNE in global structure semantic extraction. The experimental results on four real-world datasets show that our proposed model outperforms the other competitive baselines in terms of AUROC and AUPRC.

The rest of this paper is organized as follows. Section 2 provides an overview of existing relation assessment methods. In Section 3, we introduce some necessary definitions that will be used in this paper and formulate the relation prediction task for dynamic heterogeneous networks. Section 4 describes our proposed model in detail. Extensive experimental results and analyses are presented in Section 5. Finally, we conclude this paper in Section 6. The main notations used in this paper are listed in Table 1.

## 2. Literature Review

In this section, we briefly review the research development trends concerning relation prediction and divide the approaches into two categories, including traditional heuristic methods and graph representation learning methods.

The traditional heuristic methods for relation prediction, such as common neighbors [22], Jaccard coefficient [23], Adamic Adar (AA) [24], resource allocation (RA) [25] and preferential attachment (PA) [26], mainly exist in static homogeneous networks where nodes and edges are of the same type. These methods attempt to quantify neighborhood overlap between nodes, while minimizing deviations caused by degrees. The basic idea of heuristic-based methods is typically based on the measures of nodes similarity. In general terms, the more similar the two nodes are, the more likely to have a link between them. For instance, both RA index and AA index attach more importance to common neighbor nodes in low degrees, which intuitively provide more information than common neighbor nodes in high degrees. Albeit simple, these strategies can still achieve competitive performance compared with other methods in a specific scenario.

The idea of graph representation learning is to extract the latent network features from the complicated topological structure and to encode features, such as node embedding vectors in a low-dimensional space. For a relation prediction task, the learned hidden features should preserve network properties in local-wise and global-wise, so that the unknown links can be accurately predicted. However, most graph representation learning methods focus on the topological properties of nodes (e.g., in-degree or out-degree, random walk distance, first-order proximity and so on). Early solutions [13,14] learned the hidden representations of vertices by deploying random walk in the static graph, which samples the first- and second-order similarity of nodes. Subsequently, node2vec [15] made some innovations in generating the random walk strategy, which combines depth-first sampling (DFS) and the breadth-first sampling (BFS), while efficiently exploring spatial contextual information in graph data. Ribeiro et al. [27] proposed struc2vec, which measures the structural similarity from a global perspective without requiring two nodes to be nearby. GraphSAGE [28] sampled a certain number of neighborhoods for further aggregation. Graph attention network (GAT) [29] utilized the self-attention mechanism to learn the weighting function for neighborhood matching.

Considering that networks based on UGC in real life normally vary over time, which exhibits dynamic evolution characteristics, several approaches were proposed to capture the temporal features. A straightforward way of modeling dynamic network is to split it into snapshot sequences along the timeline, which discretizes the continuous changes. Goyal et al. [30] proposed the dyngraph2vec, which learns the underlying network dynamics of evolution by constructing a deep recurrent architecture. Similarly, [31,32] adopted the long short-term memory network (LSTM) to learn the temporal dependencies of all dynamic network snapshots. Such recurrent methods capture temporal features through selective memory and forgetting mechanism which enables the model to handle long sequences. However, the shortage of these methods is obvious, which depend heavily on the time span of aggregated snapshot. Different from the above methods, researchers in DySAT [33] extract the structural information and dynamics node embedding simultaneously, via a self-attention mechanism. EvolveGCN [34] adapted the graph convolutional network (GCN) throughout the time dimension and utilized an RNN architecture to update the GCN parameters.

Furthermore, with the increasing diversity of the real-world, user-generated network, the nodes and edges in networks have gradually developed from a single type to a mixture of multiple types, which shows multi-source heterogeneous characteristics. The heterogeneous graph neural network (HetGNN) [35] conducted heterogeneous graph embedding by gathering the nodes with same type via a correlated sampling. Dong et al. [36] developed two metapath-based representation learning methods, namely metapath2vec, and its variant metapath2vec++, which learn the topological and semantic correlations in heterogeneous networks. Cen et al. [37] divided a node embedding procedure into two portions, namely the base embedding and the edge embedding, which share parameter information across different edge types, allowing the extraction of heterogeneous information.

Currently, limited attempts have been made to investigate the embedding of dynamic heterogeneous networks. HA-LSTM [17] firstly employ GCN to extract structural features from heterogeneous information network, then leverage a broad learning- and attention-based structure to capture the dynamic changes over timeline. DHNE [18] constructed a historical–current network structure with consecutive snapshots to capture temporal dependencies in the dynamic heterogeneous network, then a metapath-based random walk was conducted to extract intricate semantic information. The DyHATR [19] model built a hierarchical attention model to better learn the heterogeneity of static snapshots and captured the temporal evolution patterns via an attentive RNN structure. Nevertheless, none of the preceding techniques have established a multi-view embedding strategy, instead of focusing on the localized features while neglecting the global characteristics.

## 3. Preliminaries

In this section, we formulate the relation prediction problem for dynamic heterogeneous network. Firstly, we introduce some definitions and necessary notations that will be used in this paper, as follows:

**Definition** **1** (Dynamic heterogeneous network). *A dynamic heterogeneous network (DyHN) can be represented as a set of observed graph, $G=\left\{G^{1},G^{2},\cdots ,G^{T}\right\}$, which contains T snapshots. $G^{t}=\left(V^{t},E^{t},F,\varphi \right)$, with a adjacency matrix $A^{t}$, denotes the snapshot at time t, where $V^{t}$ is the set of nodes and $E^{t}$ is the set of edges. $F:V^{t}\to T$ denotes the mapping function for node types, while $\varphi :E^{t}\to R$ is the edge type mapping function. For a dynamic heterogeneous network, the following conditions must be satisfied that $\left|T\right|+\left|R\right|>2$.*

**Definition** **2** (Dynamic heterogeneous network representation learning). *Given a dynamic heterogeneous network, $G=\left\{G^{1},G^{2},\cdots ,G^{T}\right\}$, the objective of graph representation learning is to encode the node as a low-dimensional vector that contains graph structure and local neighborhood information, that is, mapping snapshot $G^{t}$ into a hidden space, $Z^{t}\in R^{\left|V^{t}\right|\times d}$, where d is the final embedding dimension.*

**Definition** **3** (Relation prediction). *Given a series of observed network snapshots, $G=\{G^{1},G^{2},$$\cdots ,G^{T}\}$, relation prediction tasks in dynamic heterogeneous network can be viewed as a prediction on an evolving network with multiple types of nodes and edges. Besides, each snapshot is considered as a static heterogeneous network in this work, the link connection at time stamp, t+1, is determined by the spatial features and temporal evolutionary trajectory extracted from historical snapshots.*

## 4. Community-Aware Dynamic Heterogeneous Network Embedding Method

In this section, we propose a community-aware dynamic heterogeneous network embedding method. In Section 4.1, we derive heterogeneous properties guided by a Markov-chain-optimized metapath. For the sake of computation efficiency, we perform negative sampling for learning node embedding in the skip-gram model. Then, we aggregate subgraph-level features using the edge-centric attention mechanism in Section 4.2, which preserves the low-order proximity of nodes. In Section 4.3, we extract the structure semantic of network from a macro perspective using clustering technique. To capture the temporal evolutionary patterns, we develop an encoder–decoder structure based on recurrent neural network in Section 4.4.

### 4.1. Heterogeneous Contents Encoding

**Figure 2:**
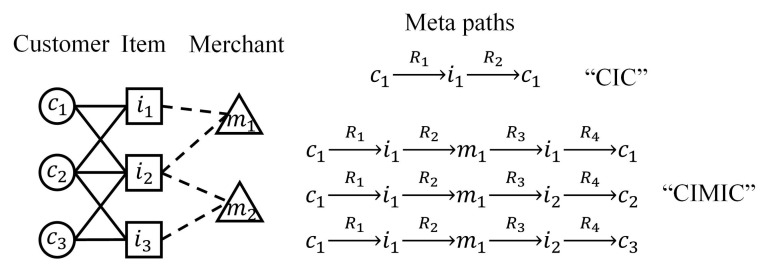
An illustrative example of a heterogeneous commodity supply–demand network guided by different metapaths.

Different from the homogeneous network, a heterogeneous network is made up of multiple types of nodes and edges. To extract the heterogeneity, inspired by metapath2vec [36], we perform random walk under the guidance of metapath on each heterogeneous network snapshot in order to capture the spatial topology and semantic information. Generally, a metapath strategy, P, can be described as a path that is predefined as: $P:T_{1}\overset{R_{1}}{\to }T_{2}\overset{R_{2}}{\to }\cdots T_{t}\overset{R_{t}}{\to }\cdots \overset{R_{n}}{\to }T_{n+1}$; therein, $R=R_{1}•R_{2}•\;\cdots \;•R_{n}$ represents the multi-level relationships between nodes $T_{1}$ and $T_{n+1}$. For illustration, consider the commodity supply–demand network shown in Figure 2, the metapath “CIC” denotes the common interest in items between two customers, and “CIMIC” represents that two customers purchase similar items from the same merchant.

The key point of conducting random walk on heterogeneous networks is to determine the transition probabilities for each step. For the sake of efficiency and effectiveness, we associate the metapath-based random walk with higher-order Markov chains [38] to facilitate the learning of nodes distribution.

**Theorem** **1.** *Given an arbitrary metapath, $P:T_{1}\overset{R_{1}}{\to }T_{2}\overset{R_{2}}{\to }\cdots T_{t}\overset{R_{t}}{\to }T_{t+1}$, there exists a k-order Markov chain if—and only if—P can be decomposed into a collection of distinct k-length metapaths, $\left\{T_{l}\overset{R_{l}}{\to }T_{l+1}\overset{R_{l+1}}{\to }\cdots \overset{R_{l+k-1}}{\to }T_{l+k}\right\}$, while satisfying the condition that current state, $T_{l+k}$, is only determined by $\left\{T_{l},T_{l+1},\cdots ,T_{l+k}\right\}$. We can then leverage the transition probabilities obtained by k-length Markov chain to guide P-concerned random walks.*

Note that the metapath decomposition mentioned above can be interpreted as a process of factor extraction. For instance, we can decompose the metapath “CIMIC” into a set of metapath factors: “CIM”, “IMI”, “MIC” and “ICI”. It is evident that the present state can only be determined by the last two states. Therefore, motivated by this, we utilize a second-order Markov chain to represent the metapaths of this “CIMIC” type and introduce the node transition probability matrix, as follows:

(1) $$M_{k|j,i}≜\left\{\begin{array}{cc} P_{T_{l+1}\|T_{l+2}} & ,F:v_{i,j,k}\to T_{l,l+1,l+2}\\ 0 & ,otherwise \end{array}\right.$$

where $M_{k|j,i}$ indicates the transition probability to node *k*, given the last hop node, *j*, and the second last hop node, *i*. F denotes the type mapping function for nodes *i*, *j* and *k*. $P_{T_{l+1}\|T_{l+2}}$ represents the transition probabilities proposed in path ranking algorithm (PRA) [39], which can be calculated as follows:

(2) $$P_{T_{l+1}\|T_{l+2}}=D_{T_{l+1}\|T_{l+2}}^{-1}A_{T_{l+1}\|T_{l+2}}$$

where $A_{T_{l+1}\|T_{l+2}}$ is the adjacency matrix between nodes in type $T_{l+1}$ and nodes in type $T_{l+2}$, and $D_{T_{l+1}\|T_{l+2}}$ is the degree matrix. Thus, given a dynamic heterogeneous network snapshot, $G^{t}=\left(V^{t},E^{t},F,\varphi \right)$, and a meta path scheme, $P:T_{1}\overset{R_{1}}{\to }T_{2}\overset{R_{2}}{\to }\cdots T_{t}\overset{R_{t}}{\to }T_{t+1}$ satisfies the second-order Markov chain. The transition probability at step *t* is defined as follows:

(3) $$Prob\left\{X_{t}=k|X_{t-1}=j,X_{t-2}=i,P\right\}≜M_{k|j,i}$$

To ease exposition, we abbreviate term $Prob\left\{\cdot \right\}$ as $p\left(\cdot \right)$. Subsequently, we utilize the skip-gram to learn node representations on the dynamic heterogeneous networks snapshot by maximizing the probability of the existence of heterogeneous neighbor nodes. After given a dynamic heterogeneous network snapshot $G^{t}$ with |T| types of nodes and the neighborhood sampling corpus, $V_{P}$, guided by metapath P, we define the objective function as follows:

(4) $$arg\max_{\theta }\sum_{i\in V_{P}}^{}\sum_{c\in T}\sum_{m\in N_{c}\left(i\right)}\log p\left(m|i;\theta \right)$$

where $N_{c}\left(i\right)$ denotes the neighborhoods of node *i* with type *c*, and θ is the set of parameters. Generally, the transition probability $p\left(m|i;\theta \right)$ is normalized by the softmax function [18,36,40].

(5) $$p\left(m|i;\theta \right)=\frac{\exp\left(X_{c_{m}}^{t}\cdot X_{i}^{t}\right)}{\sum_{m^{\prime }\in V_{P}}\exp\left(X_{m^{\prime }}^{t}\cdot X_{i}^{t}\right)}$$

where $X_{c_{m}}^{t}$ is the context vector of *m* and $X_{i}^{t}$ indicates the embedding of node *i*.

To relieve the burden of calculation, we deploy a negative sampling, the same as [40], which yields great performance in practice. We firstly define a negative sample size, *N*, then the final objective function is shown as follows:

(6) $$\log\sigma \left(X_{c_{m}}^{t}\cdot X_{i}^{t}\right)+\sum_{n=1}^{N}E_{u_{n}∼P\left(u\right)}\left[\log\sigma \left(-X_{u_{n}}^{t}\cdot X_{i}^{t}\right)\right]$$

where $\sigma \left(\cdot \right)$ is the sigmoid function that limits the value to a range of $\left[0,1\right]$, $P\left(u\right)$ is the predefined sampling distribution and $u_{n}$ means node *u* has been negative sampled for *n* times. The stochastic mini-batch gradient descent (SMGD) algorithm is utilized to optimize the objective function, which reduces the computational overhead and randomness while maintaining a fast convergence rate.

### 4.2. Subgraph-Level Feature Aggregation

After conducting metapath-based random walk on dynamic heterogeneous snapshots, we obtain the node-centric embedding, while the heterogeneous network also contains different types of edges. In order to incorporate edge-wise heterogeneous information to each node representation, we introduce edge-centric embedding method, including embedding for the same types of edges and different types of edges, with attention mechanism. In general, we perform a subgraph-level aggregation operation to obtain the representation of each snapshot in a micro perspective.

**Figure 3:**
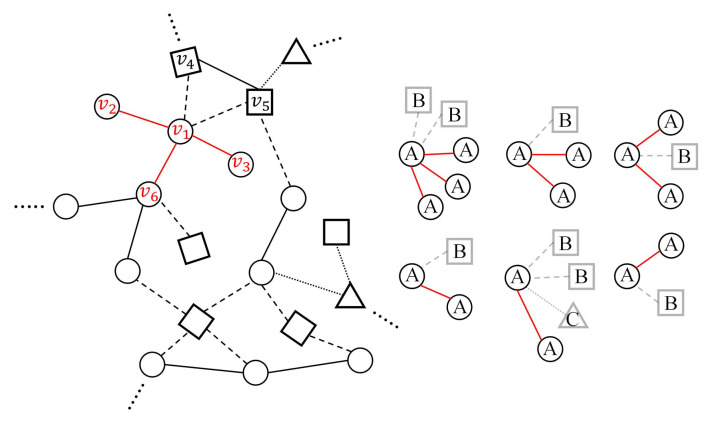
The schematic diagram of calculating the weight coefficient of edge based neighbors for the middle node.

For the edge in same type, the edge-centric attention model aims to learn the importance coefficient of each node’s neighborhoods in same type and aggregate these features to generate hidden representations. Taking Figure 3 as an example, there are five neighbors around the node $v_{1}$, while node $v_{2}$, $v_{3}$ and $v_{6}$ are the same type as node $v_{1}$. Therefore, this step involves handling with the set of nodes $\left\{v_{1},v_{2},v_{3},v_{6}\right\}$.

Suppose the input features consist of node embedding of each snapshot, $G^{t}\in G$; we deploy edge-centric attention model for each node pair with the same edge type. The importance coefficient of node $j\in N_{i_{r}}$ to node *i* with edge type *r* and *t*-th snapshot can be formulated as follows:

(7) $$w_{{\left(i:j\right)}_{r}}^{t}=\frac{\exp\left(\sigma \left(\alpha_{r}^{T}\left[W_{r}X_{i}^{t}⊕W_{r}X_{j}^{t}\right]\right)\right)}{\sum_{u\in N_{i_{r}}^{t}}\exp\left(\sigma \left(\alpha_{r}^{T}\left[W_{r}X_{i}^{t}⊕W_{r}X_{u}^{t}\right]\right)\right)}$$

where $N_{i_{r}}^{t}$ denotes the neighbors of node *i* with type *r* at *t*-th snapshot, and $W_{r}$ is the learnable parametric matrix. $\sigma \left(\cdot \right)$ is the LeakyReLU activation function. $X_{i}^{t}$, $X_{j}^{t}$ and $X_{u}^{t}$ are the embedding for node *i*, *j*, and *u* at *t*-th snapshot, respectively, and ⊕ represents the concatenation operation. Then, we aggregate the features of neighbors with same type edge by employing nonlinear transformation, as follows:

(8) $${\tilde{X}}_{i_{r}}^{t}=\sigma \left(\sum_{j\in N_{i_{r}}^{t}}w_{{\left(i:j\right)}_{r}}^{t}\cdot W_{r}X_{j}^{t}\right)$$

where ${\tilde{X}}_{i_{r}}^{t}$ is the aggregated embedding of node *i* for the same type of edges at *t*-th snapshot. Here, $\sigma \left(\cdot \right)$ is the tanh function.

Subsequently, we further explore the impact of edge-type-based neighbors for certain nodes. Firstly, we carry out the feature transformation that map aggregated node embedding into high-level space $\sigma \left(W_{e}\cdot {\tilde{X}}_{i_{r}}^{t}+b_{e}\right)$ through a nonlinear function, $\sigma \left(\cdot \right)$, such as ReLU, tanh and sigmoid. $W_{e}$ and $b_{e}$ are the learnable parametric matrix and bias vector, respectively. Then, we measure the influence of different types of edges to a specific node by implementing an edge-centric attention model. The weight coefficient of node *i* with edge type *r* at *t*-th snapshot $\beta_{i_{r}}^{t}$ is normalized by the softmax function:

(9) $$\beta_{i_{r}}^{t}=\frac{\exp\left(q^{T}\cdot \sigma \left(W_{e}\cdot {\tilde{X}}_{i_{r}}^{t}+b_{e}\right)\right)}{\sum_{r\in R}\exp\left(q^{T}\cdot \sigma \left(W_{e}\cdot {\tilde{X}}_{i_{r}}^{t}+b_{e}\right)\right)}$$

where *q* is the attentive parameterized vector and tanh function is utilized as activation function. With the normalized attention weights, we can finally obtain the embedding for node *i* at *t*-th snapshot from different edge-levels, which can be expressed as follows:

(10) $${\tilde{X}}_{i}^{t}=\sum_{r=1}^{\left|R\right|}\beta_{i_{r}}^{t}\cdot {\tilde{X}}_{i_{r}}^{t}$$

After obtaining the representation of each node in the snapshot, the overall node embedding at *t*-th snapshot can be described as follows: $Z_{micro}^{t}=\left\{{\tilde{X}}_{1}^{t},{\tilde{X}}_{2}^{t},\cdots ,{\tilde{X}}_{i}^{t},\cdots ,{\tilde{X}}_{\left|V^{t}\right|}^{t}\right\}$, where ${\tilde{X}}_{i}^{t}\in R^{F}$, $F\ll \left|V^{t}\right|$ is the number of feature embedding dimensions.

### 4.3. Community-Level Semantic Learning

The local structure of nodes is crucial for dynamic heterogeneous network representation, while the global structure also plays an important role in portraying the network. Community structure exists in many real-world networks, whether they are homogeneous or heterogeneous, which reflect the global structure of networks in a macroscopic perspective. Motivated by this intuition, we present a community-aware graph embedding method with network clustering technique for extracting the structure semantic in community-level, which encode node information into low-dimensional representations.

However, in dynamic heterogeneous networks, community structures are generally regarded as a priori information, which can not be known in advance. Following the work DEC [41], we first initialize a set of *K* cluster centroids ${\left\{c_{j}\right\}}_{j=1}^{K}$ by a random selection procedure. The clustering objective function is defined as a Kullback–Leibler divergence (KL) loss between the soft probability distribution, *Q*, and the auxiliary probability distribution, *P*, which can be expressed as follows:

(11) $$L_{c}=KL\left(P∥Q\right)=\sum_{i}\sum_{j}p_{ij}\log\frac{p_{ij}}{q_{ij}}$$

where $q_{ij}$ can be interpreted as the probability that measures the similarity between node *i* and cluster center *j* by Student’s *t*-distribution, as follows:

(12) $$q_{ij}=\frac{(1+∥{\tilde{X}}_{i}^{t}-c_{j}{{∥}^{2}/n)}^{-\frac{n+1}{2}}}{\sum_{j^{\prime }\in K}{\left(1+∥{\tilde{X}}_{i}^{t}-c_{j^{\prime }}{∥}^{2}/n\right)}^{-\frac{n+1}{2}}}$$

where *n* denotes the degree of freedom of the Student’s *t*-distribution, and ${\tilde{X}}_{i}^{t}$ is the micro node embedding of node *i* generated in Section 4.2. Empirically, we set n=1 in our experiments. $p_{ij}$ is the auxiliary probability distribution calculated by the following:

(13) $$p_{ij}=\frac{q_{ij}^{2}/\sum_{i}q_{ij}}{\sum_{j^{\prime }\in K}q_{ij^{\prime }}^{2}/\sum_{i}q_{ij}}$$

During backpropagation, the stochastic gradient descent algorithm is utilized to iteratively optimize the cluster loss function so as to bring the node closer to its cluster centroid. The partial derivative of $L_{c}$ with respect to variables ${\tilde{X}}_{i}^{t}$ and $c_{j}$ are shown as follows:

(14) $$\frac{\partial L_{c}}{\partial {\tilde{X}}_{i}^{t}}=\frac{n+1}{n}\sum_{j=1}^{K}{\left(1+\frac{∥{\tilde{X}}_{i}^{t}-c_{j}{∥}^{2}}{n}\right)}^{-1}\cdot \left(p_{ij}-q_{ij}\right)\left({\tilde{X}}_{i}^{t}-c_{j}\right)$$

(15) $$\frac{\partial L_{c}}{\partial c_{j}}=\frac{n+1}{n}\sum_{i=1}^{\left|V^{t}\right|}{\left(1+\frac{∥{\tilde{X}}_{i}^{t}-c_{j}{∥}^{2}}{n}\right)}^{-1}\cdot \left(p_{ij}-q_{ij}\right)\left(c_{j}-{\tilde{X}}_{i}^{t}\right)$$

After sending the gradients down to update the parameters, we can obtain the node embedding, $Z_{macro}$, and cluster centroids, $c_{j}$, from a macro perspective, where $Z_{macro}\in R^{\left|V^{t}\right|\times d}$ is constructed by updated ${\tilde{X}}_{i}^{t}$, and *d* is the dimension of the embedded features.

**Figure 4:**
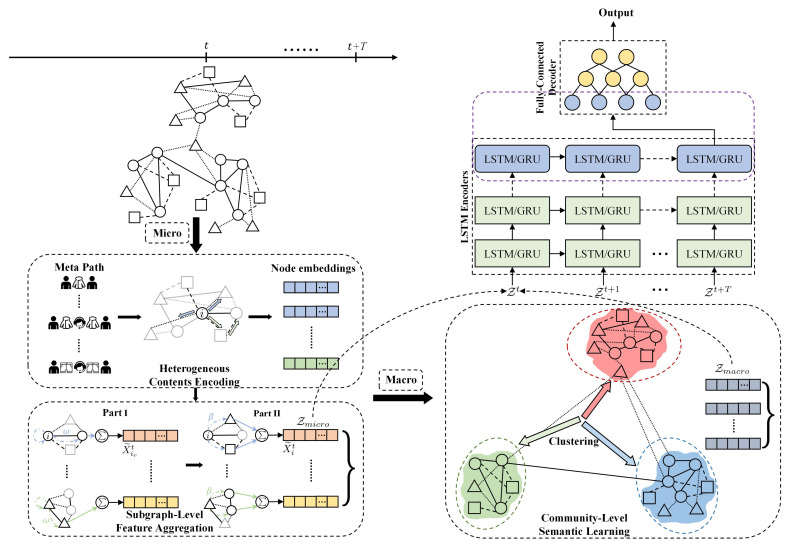
The overall architecture of the proposed CDHNE. At the *t*-th snapshot, CDHNE extracts micro node representations and macro structure semantic sequentially, and fuse them with parameterized weights as the input of temporal dynamic extraction model. The changes of entities and semantics in DyHN are captured with RNN-based model due to its superiority in handling long sequences. Finally, the output are transformed to the probability distribution through the fully-connected decoder.

Finally, we construct the ultimate node embedding using a combination of the micro node embedding $Z_{micro}$ and macro node embedding $Z_{macro}$, which is to model the overall structure of a dynamic heterogeneous network. Specifically, the overall node embedding $Z^{t}$ at *t*-th snapshot is defined as follows:

(16) $$Z^{t}=\lambda Z_{micro}^{t}+\left(1-\lambda \right)Z_{macro}^{t}$$

where λ denote the trade-off parameter that balances the weight of the micro and macro node embedding at ultimate node representations. The overall framework of CDHNE is presented in Figure 4.

### 4.4. Temporal Evolutionary RNN Model

One of the major characteristics of dynamic heterogeneous networks is the time-varying characteristic. There are many scenarios of changing network structures, such as establishing citation relationships between authors, product recommendations between users, those newly added or removed UGCs, and so forth. For this reason, we propose two variations of our method, as presented in Figure 4: (i) CDHNE-GRU, (ii) CDHNE-LSTM. Two high-profile, RNN-based models—gated recurrent unit (GRU) and long short-term memory (LSTM)—are leveraged in our proposed method with encoder–decoder structure to enable the capability of capturing a network’s evolutionary patterns and to further extract comprehensive information along continuous snapshots.

Gated recurrent unit is a modification to the RNN hidden layer that makes it much better for capturing long-range connections and helps a lot with the vanishing gradient problems. Through the above structural semantic learning and feature aggregation operation, we can finally obtain the node embedding for all snapshots, $\left\{Z^{1},Z^{2},\cdots ,Z^{T}\right\}$, where $Z^{t}\in R^{\left|V^{t}\right|\times d}$ and $\left|V^{t}\right|$ denote the number of nodes at *t*-th snapshot, and *d* is the size of the embedded dimension. Then, we take these embeddings as the input of GRU. These following are the equations that govern the computation of a GRU unit:

(17) $$\begin{array}{cc} \Gamma_{u}^{t} & =\sigma \left(W_{u}\left[c^{t-1}⊕Z^{t}\right]+b_{u}\right)\\ \Gamma_{r}^{t} & =\sigma \left(W_{r}\left[c^{t-1}⊕Z^{t}\right]+b_{r}\right)\\ {\tilde{c}}^{t} & =\tanh(W_{c}\left[\left(\Gamma_{r}*c^{t-1}\right)⊕Z^{t}\right]+b_{c})\\ c^{t} & =\Gamma_{u}*{\tilde{c}}^{t}+\left(1-\Gamma_{u}\right)*c^{t-1}\\ a^{t} & =c^{t} \end{array}$$

where $\Gamma_{u}$, $\Gamma_{r}\in R^{F}$ denote the update and reset gates, respectively. $c^{t}$, ${\tilde{c}}^{t}$ are the memory unit and the candidate value, respectively. $W_{u}$, $W_{r}$, $W_{c}\in R^{F\times 2d}$ and $b_{u}$, $b_{r}$, $b_{c}\in R^{F}$ are trainable parameters. *F* is the dimension of output embedding. σ(·) is the activation function. ⊕ indicates the concatenation operation, and * is the the operation of Hadamard product.

Compared with the GRU, the LSTM model achieves better representation and introduces the forget gate, $\Gamma_{f}^{}$, for controlling the information of previous moments more independently. The formulations of the single LSTM network at *t*-th snapshot are shown as follows:

(18) $$\begin{array}{cc} \Gamma_{u}^{t} & =\sigma \left(W_{u}\left[a^{t-1}⊕Z^{t}\right]+b_{u}\right)\\ \Gamma_{f}^{t} & =\sigma \left(W_{f}\left[a^{t-1}⊕Z^{t}\right]+b_{f}\right)\\ \Gamma_{o}^{t} & =\sigma \left(W_{o}\left[a^{t-1}⊕Z^{t}\right]+b_{o}\right)\\ {\tilde{c}}^{t} & =tanh(W_{c}\left[a^{t-1}⊕Z^{t}\right]+b_{c})\\ c^{t} & =\Gamma_{u}*{\tilde{c}}^{t}+\Gamma_{f}*c^{t-1}\\ a^{t} & =\Gamma_{o}^{t}*\tanh\left(c^{t}\right) \end{array}$$

where $\Gamma_{u}^{t}$, $\Gamma_{f}^{t}$, $\Gamma_{o}^{t}\in R^{F}$ denote the update, forget and output gate, respectively. State vector $a^{t}$ is the element-wise product of the output gate, $\Gamma_{u}^{t}$, and the memory unit, $c^{t}$. $W_{u}$, $W_{f}$, $W_{o}$, $W_{c}\in R^{F\times 2d}$ and $b_{u}$, $b_{f}$, $b_{o}$, $b_{c}\in R^{F}$ are trainable parameters. The other notations represent the same meaning as the GRU model.

As depicted in Figure 4, in order to achieve a more effective temporal evolution learning, we stack several LSTM networks to construct a multi-layer structure. In each layer, there can be *T* recurrent memory units arranged like a chain, which is to deliver parameters to next timestamp. Firstly, we encode the input node embedding into hidden representation via an RNN-based model, then apply several fully connected layers as the decoder for the final relation prediction between two nodes.

### 4.5. Complexity Analysis of CDHNE

In this work, dynamic heterogeneous networks are represented by *T* static snapshots. Therefore, for each snapshot, we mainly consider the time complexity on two stages. Firstly, for the subgraph-level information extraction, which consists of heterogeneous contents learning and feature aggregation. During the metapath-based random walk, given a metapath set P with $\left|V^{t}\right|$ nodes, walk length, *l*, and walks per node, *w*, the time complexity is $O(dw\left|V^{t}\right|l^{2})$, in which *d* is the embedding dimension. In the process of calculating node embeddings, the theoretical time complexity of skip-gram is extremely high; we employ negative sampling for reducing complexity as much as possible. During the feature aggregation stage, the time complexity is $O(dl{\left|R\right|}^{2}\left|E^{t}\right|)$, where $\left|R\right|$ is the number of edge types, and $\left|E^{t}\right|$ is the number of edges at *t*-th snapshot.

For the community-level semantic learning, in each snapshot, the time complexity of calculating node embedding in macro perspective can be divided into two parts. To calculate the probability distribution $q_{ij}$ and $p_{ij}$, our method takes $O\left(dK\left|V^{t}\right|\right)$ and $O\left(K\left|V^{t}\right|\right)$, respectively, where *K* is the initial number of cluster centroids. The other is $O\left(dK\left|V^{t}\right|\right)$, in calculating the gradient of parameters ${\tilde{X}}_{i}^{t}$ and $c_{j}$ simultaneously. Since $d\ll \left|V^{t}\right|$, the time complexity of this part is almost linear with the number of nodes.

Besides, for the extraction of temporal evolution patterns, we utilize the RNN-based model, of which the time complexity is normally related to hardware execution. Thus, we introduce the model complexity in this subsection. The number of parameter for each cell in the LSTM model is $4*\left(dn\left|V^{t}\right|+n+n^{2}\right)$, where *n* denotes the size of output term. Finally, the pseudocode for CDHNE is shown in Algorithm 1.
**Algorithm 1** The CDHNE algorithm**Input:** A dynamic heterogeneous network G with *T* snapshots, the predefined embeddingdimension *d*.**Output:** The probability of the linkage between two nodes1:**for** each snapshot $G^{t}\in \left\{G^{1},G^{2},\cdots ,G^{T}\right\}$ **do**2:   $X_{i}^{t}\leftarrow$ A Markov-chain-optimized metapath random walk3:   **for** each edge in specific type r∈R **do**4:     Calculate the importance weight $w_{{\left(i:j\right)}_{r}}^{t}$ of node pair $\left(i,j\right)$ by Equation (7)5:     Aggregate the features of neighbors in same type and obtain the type-specific node embedding ${\tilde{X}}_{i_{r}}^{t}$6:   **end for**7:   Calculate the weight coefficient $\beta_{i_{r}}^{t}$ of node *i* with edge type *r* at *t*-th snapshot by Equation (9)8:   Obtain the node representation at *t*-th snapshot $Z_{micro}^{t}$ in a micro perspective through feature aggregation with different type of edges9:   Obtain the community-level semantic information $Z_{macro}$ by a clustering process10:**end for**11:Generate comprehensive node embedding $Z^{t}$ for each snapshot by Equation (16)12:Get the probability of the link existence through GRU/LSTM encoder–decoder structure

## 5. Experiments and Result Analysis

In this section, we evaluate the performance of the proposed model on four real-world datasets. We firstly introduce the datasets and the configuration of the experimental environment. Then, we introduce the baselines in detail. We also conduct elaborate experiments for relation prediction tasks and demonstrate the effectiveness of main components. Moreover, we analyze the influence of sampling granularity and investigate the sensitivity of hyper-parameters. The detailed statistics of datasets are summarized in Table 2. (https://github.com/ZijianChen1998/CDHNE.git) The implementation of our CDHNE model are publicly accessed on 8 February 2022.

### 5.1. Experiment Setup and Dataset Description

We abstract various user-generated contents as heterogeneous nodes and select four dynamic heterogeneous networks covering academic, commerce and social interaction fields as our experimental datasets. The detailed description are presented as follows:

- **DBLP** [42] (https://dblp.uni-trier.de) (15 December 2021): The DBLP dataset comprises academic literature information in the field of computer science. In this experiment, we adopt a subset of the DBLP dataset collected by [18], and compress the information into 19 snapshots, which contains 3 types of nodes, i.e., authors, papers and venues.
- **AMiner** [43] (https://www.aminer.cn/data) (15 December 2021): AMiner is a big data mining and service system platform which helps researchers to mine rich academic information. In this paper, we use the evolved dynamic heterogeneous network released by [18], which establishes the relationships among authors, articles and conferences.
- **EComm** (https://tianchi.aliyun.com/competition/entrance/231721) (15 December 2021): The EComm dataset was launched in CIKM-2019 E-Commerce AI Challenge, which records the consumers’ shopping behavior over an 11-day period from 10 June 2019, to 20 June 2019. It consists of three files (i.e., user behavior files, user information sheets and product information tables).
- **Math-Overflow** (https://snap.stanford.edu/data/sx-mathoverflow.html) (15 December 2021): This dataset [44] contains interactions of users over time, which are sampled from the stack exchange website Math-Overflow. There are three different types of directed edges (i.e., answer–question, comment–question, comment–answer) over a time span of up to 2350 days.

**Evaluation Metrics**: We choose two commonly used evaluation indicators, AUROC and AUPRC [45], to compare the relation prediction performance of different methods in dynamic heterogeneous networks. Among them, AUROC is the abbreviation of the area under the receiver operating characteristic curve (ROC), while AUPRC is the abbreviation of the area under the precision recall curve (PRC).

Noting that, AUROC can be interpreted as the probability of a randomly chosen missing link being ranked higher than a randomly chosen nonexistent link. Then, the AUROC can be formulated as follows:

(19) $$AUROC=\frac{n^{\prime }+0.5n^{\prime \prime }}{N}$$

where *N* denotes the number of independent processes, and there are $n^{\prime }$ times that the score of missing link is greater than the score of nonexistent link and $n^{\prime \prime }$ times when the opposite scenario occurs. Similarly, the PRC is plotted by precision–recall pairs. Precision measures the capacity of the classifier to label the missing links correctly, while the recall reflects the completeness of the model to discover all missing links. Intuitively, the closer the value of AUROC and AUPRC are to one, the more discriminative the model is.

In the task of community detection, we take modularity to measure the capacity of discovering community structure in dynamic heterogeneous networks, which does not need prior information about the ground truth. Let $C_{u}$ denotes the affiliation of node *u*. The calculation formula of modularity is defined as follows:

(20) $$Q=\frac{1}{2m}\sum_{uv}\left[A_{uv}-\frac{k_{u}k_{v}}{2m}\right]\delta \left(C_{u},C_{v}\right)$$

where *Q* denotes the modularity, the closer it is to 1, the better the effect of community division. *m* is the number of overall edges in network. $A_{uv}$ indicates the linkage between node *u* and *v*. $\delta \left(C_{u},C_{v}\right)=1$ only if node *u* and *v* belong to the same community, otherwise $\delta \left(C_{u},C_{v}\right)=0$.

During the phase of heterogeneous information processing, we employ metapath guided random walk for node representations, which based on the transition probability of *k*-order Markov chain. In dataset DBLP, we mainly consider metapaths involving *APA* (i.e., the coauthors semantic), and *APCPA* (i.e., the sharing publication on conferences from different authors). In dataset Aminer, we also pay attention to metapaths including *APA* and *APCPA*, with similar meanings. For the EComm dataset, we consider the relations between customers and items including browse, buy, add-to-cart and add as favorite. For the Math Overflow dataset, we are interested in the interactions between users, which manifests as answering, questioning and commenting.

To make a fair comparison, all experiments are conducted using the Windows(64-bit) PC with Intel Core i5-9300HF CPU 2.4 GHz, 16 GB RAM and NVIDIA GeForce GTX 1660Ti 6G GPU. The programming environment of CDHNE-GRU/LSTM are Python 3.7 and Tensorflow 1.15. The detailed experiment configurations are represented in Table 3.

### 5.2. Baseline Description

We compare our proposed model against eleven methods in four categories, including static homogeneous network embedding methods, static heterogeneous network embedding methods, dynamic homogeneous network embedding methods, and dynamic heterogeneous network embedding methods. The detailed descriptions are as follows.

#### 5.2.1. Static Homogeneous Network Embedding

- **DeepWalk** [13]: DeepWalk is a homogeneous network embedding method, which conducts random walk to learn the node representation in static network.
- **node2vec** [15]: The idea of node2vec is similar to DeepWalk, while considering the DFS and BFS neighborhoods simultaneously, thus improving the effect of network embedding.
- **GAT** [29]: Graph attention network leverages the attention mechanism to assign different weights to each neighbor, which adaptively realized the matching weights of different neighbors.
- **GraphSAGE** [28]: GraphSAGE samples the neighbor nodes of each vertex on the static homogeneous graph, then aggregates the feature information from neighbors.

#### 5.2.2. Static Heterogeneous Network Embedding

- **metapath2vec** [36]: metapath2vec performs a path determined random walk in heterogeneous network and leverages the skip-gram model to generate the node embedding.
- **metapath2vec++** [36]: metapath2vec++ improves the metapath2vec by further extracting the structural and semantic correlations in heterogeneous networks.
- **HetGNN** [35]: HetGNN is a graph neural network-based model for static heterogeneous network, which sample-correlated the neighbors for heterogeneous nodes with a restart random walk and obtained deep feature interactions through a encoding module.

#### 5.2.3. Dynamic Homogeneous Network Embedding

- **dyngraph2vec-RNN** [30]: A deep architecture with sparsely connected long short-term memory networks, which is able to learn the evolution patterns in homogeneous graph structures.
- **dyngraph2vec-AERNN** [30]: An improved version of dyngraph2vec-RNN, which leverage multiple fully connected layer to learn the initially hidden representations.
- **DySAT** [33]: DySAT obtains the node representations by jointly conducting self-attention operation to extract the structural information and temporal dynamics.

#### 5.2.4. Dynamic Heterogeneous Network Embedding

- **DHNE** [18]: A network representation learning method for dynamic heterogeneous networks, which construct historical–current networks snapshots in timelines and capture heterogeneous semantic information under the guidance of metapaths.
- **DyHATR** [19]: DyHATR utilize a hierarchical attention mechanism to learn heterogeneous information and capture network dynamic evolutional patterns via a temporal-attention-based recurrent neural network.

### 5.3. Relation Prediction

The objective of a relation prediction task for dynamic heterogeneous networks is to learn various node representations from previous *t*-th snapshots, then forecast the relation existence at $\left(t+1\right)$-th snapshots. Concretely, we take previous *t* snapshots, $\left\{G^{1},G^{2},\cdots ,G^{t}\right\}$, as inputs and feed them into the model, then we can obtain the node embedding at $\left(t+1\right)$-th snapshots, which contain rich information about the network, and thus are used to predict relations.

Abundant experiments are conducted on four datasets. For datasets DBLP and AMiner, we hide a certain percentage of edges to generate the training set, respectively. Meanwhile, due to the fact demonstrated in the previous work that significant metapaths help solving downstream tasks [46,47], we set different weights for *APA* and *APCPA* in DBLP as $\left\{0.8,0.2\right\}$. Similarly, we allocate weights in AMiner as $\left\{0.6,0.4\right\}$. As for dataset EComm, there is only one type of metapath *CI* with full weight. Moreover, metapath strategy in Math Overflow is based on homogeneous nodes due to the single type of nodes. We trained our proposed models for a maximum of 1000 epochs with Adam optimizer [48], which is built into tensorflow. Early stopping mechanism is utilized for better efficiency. Weights are initialized through Xavier uniform initialization [49]. We conducted all the experimental tests five times, independently.

Experimental results are summarized in Table 4. Overall, CDHNE achieves the best performance among the four datasets on two criteria, namely, AUROC and AUPRC. The notable improvement validates the effectiveness of our model in extracting comprehensive features from dynamic heterogeneous networks, while avoiding high computational overheads. Profiting from the appropriate encoding and aggregating for subgraph-level features, CDHNE significantly outperforms metapath2vec. We also concentrate on the representation of macro semantic information, which also lead to high practical connotation in heterogeneous network relationships mining. Specifically, our proposed model achieves highest score (0.903 for AUROC and 0.885 for AUPRC) in DBLP, which surpasses the static homogeneous baselines by an average of 14.7% and 10.9%, respectively. Besides, our CDHNE conduces to performance gains over latest dynamic heterogeneous methods DHNE and DyHATR with 14.6% and 4%, respectively, for AUROC in DBLP. However, on the Math Overflow dataset, our model performs slightly higher than static homogeneous methods, which attributes to the fact that there is only one type of node in Math Overflow, so that our heterogeneous encoding component scarcely take effects.

**Figure 5:**
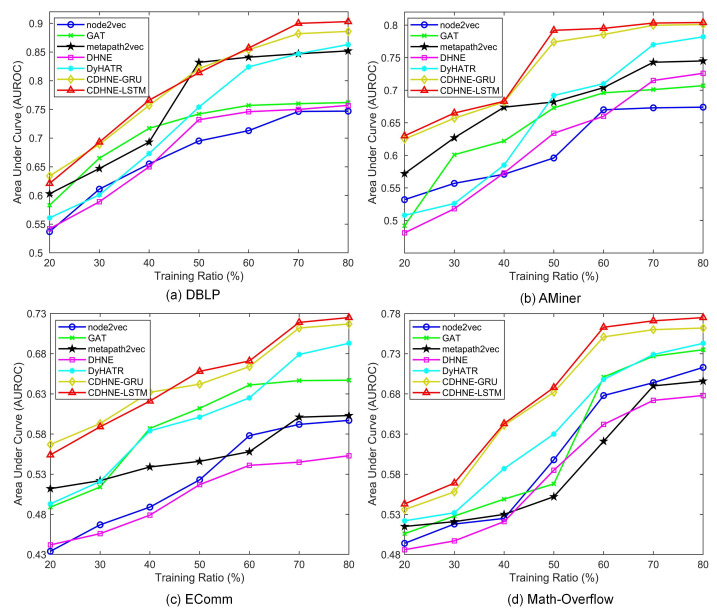
The impact of training ratio on DBLP, AMiner, EComm and Math Overflow datasets in term of area under the receiver operating characteristic curve (AUROC). Five typical baselines, i.e., node2vec, GAT, matapath2vec, DHNE and DyHATR, are selected to compare with our proposed models, which validates that CDHNE-GRU/LSTM can effectively handle relation prediction tasks regardless of the training set size.

As presented in Figure 5, we further vary the ratio of training set from 20% to 80% with the step of 10%. Five typical methods are selected for comparing the impact of different training ratio. Obviously, our model outperforms other methods, whether the training set is large or small, which validates the efficiency of CDHNE in extracting comprehensive node features. It should be noted that practically all methods perform poorly at low training ratios, while the value of AUROC grows rapidly when training ratio reaches 40% and becomes smooth at high training ratios. Thus, we can conclude that sufficient learning of node features brings advantages in handling relation prediction tasks.

### 5.4. Community Detection

To evaluate the performance of our proposed model in community structure detection, we use modularity as an assessment criteria. Since the concept of modularity was introduced, various related approaches have been proposed [50,51,52,53,54,55]. Among them, Louvain [50] attempts to discover communities by maximizing modularity with the greedy mechanism. Quick community adaptation (QCA) [52] is elaborated for tracking the evolution of community over time and updating the community structure simultaneously. Batch [51] is a batch-based incremental technique that relies on predefined strategies. GreMod [53] also performs incremental updating for capturing dynamic changes of communities. M-NMF [54] aims to preserve community structure in network embedding through matrix factorization. LBTR-SVM [55] utilized vertex classifier to affect community assignments.

**Figure 6:**
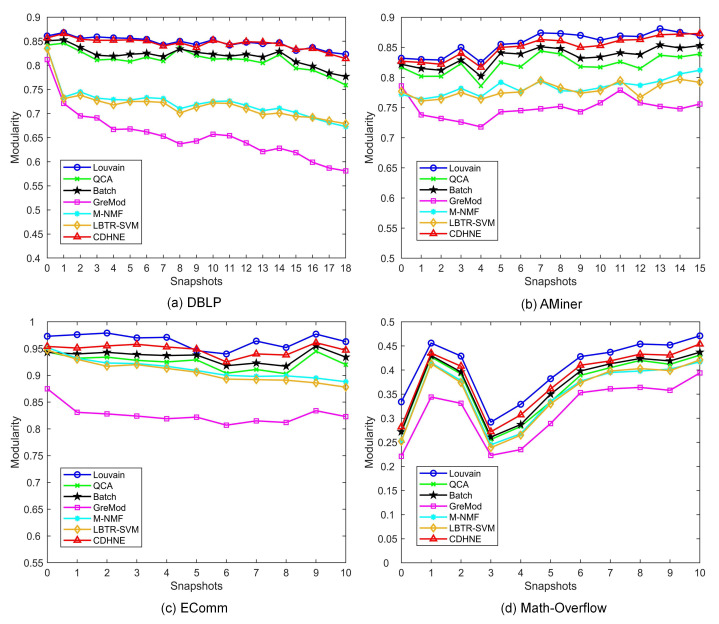
The modularity results on four real-world networks. (**a**) DBLP. (**b**) AMiner. (**c**) EComm. (**d**) Math Overflow. Six typical community detection algorithms, i.e., Louvain, QCA, Batch, GreMod, M-NMF and LBTR-SVM, are selected as competing algorithms. Observe that using the proposed CDHNE can effectively discover the community structure, while reaching a relatively high modularity compared with other methods.

**Figure 7:**
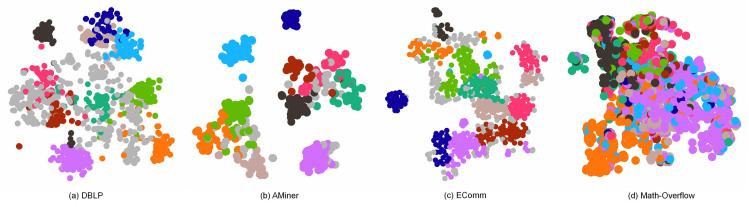
A 2D visualization of CDHNE on the first snapshot of DBLP, AMiner, EComm and Math Overflow, respectively. Here, heterogeneous nodes are depicted in the same type. Top 10 communities in scale are colored for better distinction.

Figure 6 shows the modularity comparison between six typical algorithm and our proposed model on four real-world datasets, respectively. Apparently, we can observe that CDHNE substantially outperforms the other algorithms. Specifically, CDHNE achieves on average 19%, 10.1%, 12.2% and 6.7% percent higher than GreMod on all the snapshots of DBLP, AMiner, EComm and Math Overflow, respectively, which demonstrate the superiority of our proposed model in assigning communities. Even compared with Louvain, our method still reaches only 0.2%, 0.7%, 1.6% and 2.3% percent lower on all the snapshots of DBLP, AMiner, EComm and Math Overflow, respectively. The effectiveness of CDHNE in detecting community structure comes from the pre-learning strategy, with which we first intensify the heterogeneous feature extraction at subgraph-level, then conduct clustering in the embedded space by minimizing the KL divergence. Different from capturing the tiny changes for each snapshot in a dynamic heterogeneous network, we highlight the importance of node representation learning in community detection. We visualize the first snapshot of four datasets in a low dimensional space from the learned node embedding during community-level semantic extraction. The result displayed in Figure 7 demonstrates the capacity of CDHNE in assigning communities, while embodying the drawbacks of randomly initialized centroids in handling large scale networks, according to Figure 7d.

### 5.5. Granularity of Snapshots Sampling

**Figure 8:**
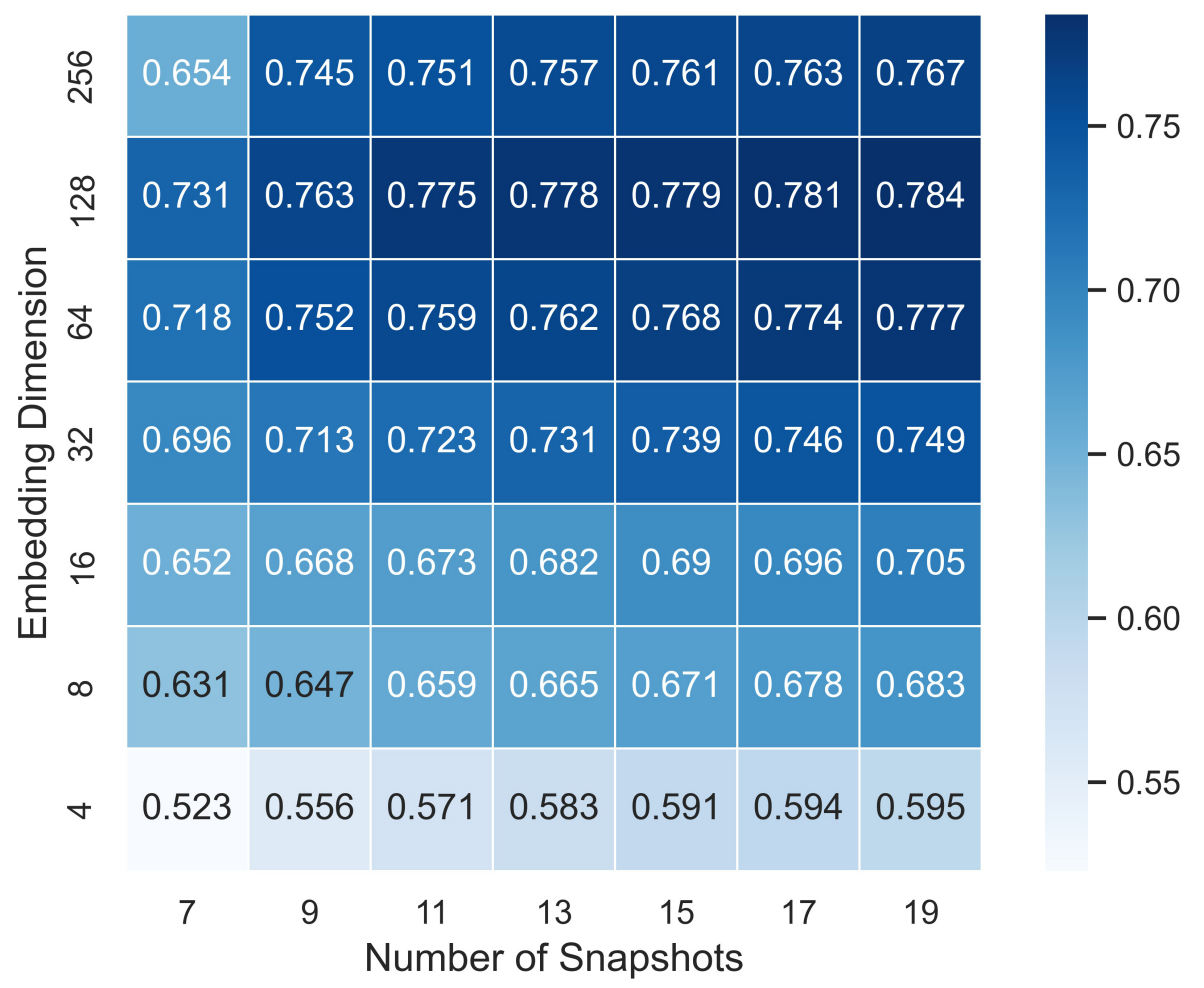
The performance of proposed model CDHNE under different sampling granularity on Math Overflow. The x-axis represents the sampling granularity, that is the number of snapshots, while the y-axis varies with the embedding dimension. The AUROC value for relation prediction task is given in each cell accompanied with a color bar on the right.

In this work, we heuristically analyze the impact of sampling granularity on relation prediction tasks, which mainly reflected in the number of snapshots. Normally, we consider the dynamic heterogeneous network as an observed series, $G=\left\{G^{1},G^{2},\cdots ,G^{T}\right\}$, which, to some extent, discretize the network at a certain sampling frequency. It is evident that different sampling granularity can bring varied effect on node representation learning, which is a problem worth pondering over. Taking Math Overflow as an example, the time span is 2350 days, which we divided into 11 snapshots in previous tasks, in which the duration of each snapshot is nearly 214 days. Figure 8 displays the experiment results of CDHNE on Math Overflow. We can observe that there exists certain regularity of AUROC variations in terms of embedding dimension and number of snapshots. Specifically, the value of AUROC grows as the number of sampled snapshots increases when the embedding dimension is fixed. The performance of CDHNE-LSTM continuously improves from 0.731 up to 0.784 in the range of $\left\{7,9,11,13,15,17,19\right\}$ snapshots under the condition that the embedded dimension is configured as 128. Thus, as the number of snapshots grows, our proposed model is capable of capturing comprehensive information over the dynamic network and perceiving more explicit information and implicit associations for predicting link existence.

In addition, we also explore the effect of the embedding dimension in node representation learning on the Math Overflow dataset. The value of AUROC keeps growing in the range of $\left\{4,8,16,32,64,128\right\}$ embedding dimension. However, the AUROC drops with embedding dimension of 256, indicating that oversize embedding dimension can cause overfitting problems. We can conclude that the finer embedding dimension and granularity are necessary ingredients in learning node representations and capturing dynamic patterns.

### 5.6. Sensitivity of Hyper-Parameters

**Figure 9:**
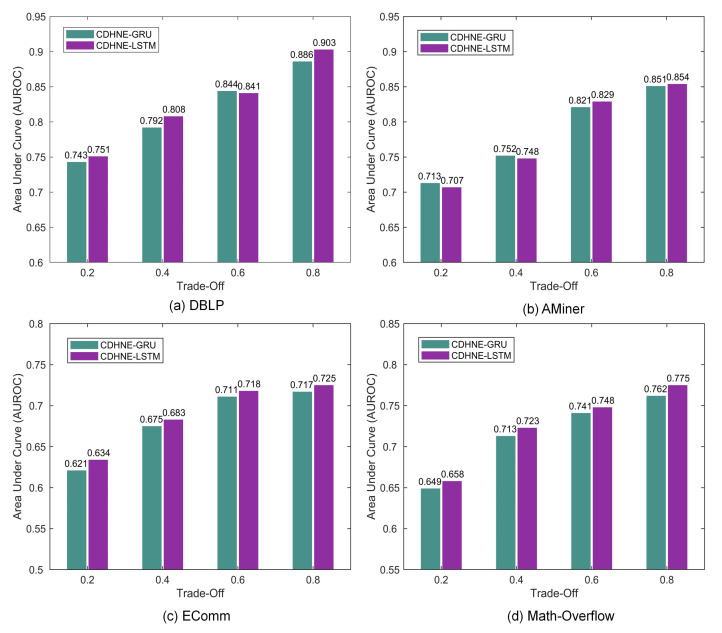
The AUROC on four datasets with respect to the trade-off λ between micro and macro node embedding. CDHNE-GRU and CDHNE-LSTM mean the two variants of our model use gated recurrent unit and long short-term memory as temporal dynamic encoder, respectively.

In this section, we investigate the effect of different hyper-parameter setting on relation prediction tasks. We evaluate two variants of our proposed model, i.e., CDHNE-GRU and CDHNE-LSTM, which differ in capturing temporal dynamics, on four real-world datasets. As Figure 9 shows, the value of trade-off λ between micro and macro node embedding influences the performance of our model, which measures the importance of micro and macro node embedding. All models are consistently reinforced with larger value of λ and reach highest AUROC at λ=0.8, which indicates the significance of micro node embedding involving localized heterogeneous contents. Interestingly, we find that the value of AUROC drops significantly when λ decreases from 0.4 to 0.2 on Math Overflow compared with other datasets. Due to the fact that the node types in the Math Overflow are single, while the heterogeneity that is mainly reflected in the types of edges and its community structure is not obvious according to previous analysis, thereby performing relatively poorly under this circumstance.

**Figure 10:**
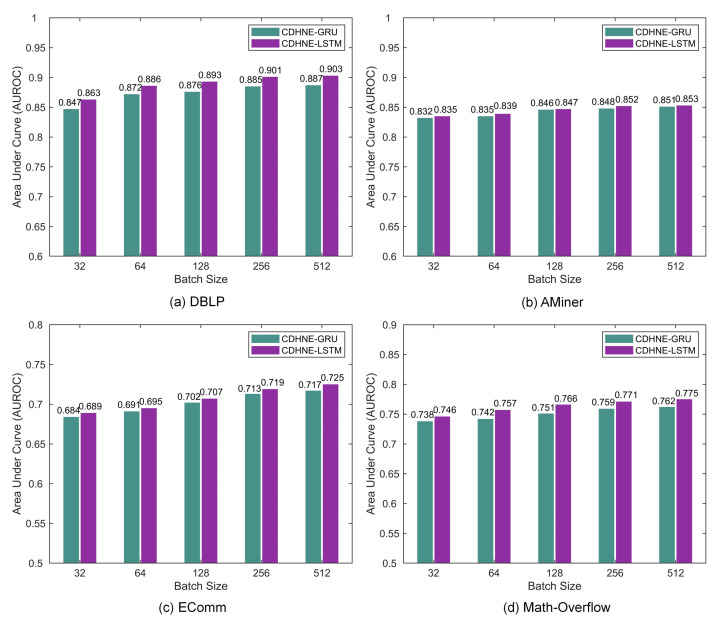
The AUROC on four real-world datasets with respect to the training batch size.

Moreover, we further explore the effect of different batch size during the model training phase. Figure 10 shows that with more data added in one batch, the performance of two variants fluctuate slightly in a uptrend, accelerating the training speed and increasing the parallelism. The best AUROC achieved by CDHNE-LSTM in DBLP, AMiner, EComm and Math Overflow are 0.903, 0.854, 0.725 and 0.775, respectively. The investigation of hyper-parameters helps us to find the best setting of our models.

## 6. Conclusions

In this paper, we abstract the source of user-generated content into a dynamic heterogeneous network and present a novel graph representation learning method, named community-aware dynamic heterogeneous network embedding, for assessing complicated relations in whole graph, abbreviated as CDHNE. Our proposed model mainly consists of three components for micro, macro node representation learning and for capturing evolutionary patterns, respectively. Based on the Markov-chain-optimized metapath, our model is able to learn the heterogeneous contents using skip-gram model. The procedure of edge-centric attention makes features aggregation at the subgraph level. We further explore the latent community structure through the clustering technique and obtain the node embedding from a macro perspective. Using an intuitive aggregation mechanism, these two parts jointly incorporate both graph structure and heterogeneous side information (e.g., node and edge features). Ultimately, we present two variants of our model with different recurrent memory unit, i.e., CDHNE-GRU and CDHNE-LSTM, for dynamics learning. Our experimental analysis shows that the well-learned useful and discriminative network information, resulting in an omnipotent representation space, leads to the effectiveness of our proposed model in various downstream tasks, such as relation assessment and community detection, compared with other state-of-the-art methods. Moreover, the visualization of CDHNE on four datasets highlights its validity in network global information extraction. The stable and competitive performance also shows the reliability of our model while under different sampling granularity.

However, there are also some problems and salient drawbacks in our work. Our proposed model is de facto a combination of graph representation learning with sequence models. The graph embedding techniques captures the heterogeneous node information from multiple perspectives, while the sequence model captures the long-term dependencies within network evolution. This solution converts the dynamic network into a static network, which enables the use of various techniques for static networks, while enlarging the potential loss of information. Further research is needed in three main directions. Firstly, we will concentrate more on digging continuous time information with less information loss. Secondly, theoretical work is needed to analysis the stability of the CDHNE to network perturbations. Finally, the implementation of our method will be reconstructed in an end-to-end way.

## Figures and Tables

**Table 1:** Summary of Main Notations.

Notation	Description
G	the set of observed network snapshots
$G^{t}$	the network snapshot at *t*-th snapshot
$A^{t}$	adjacency matrix of $G_{t}$
$V^{t}$	the set of nodes at *t*-th snapshot
$E^{t}$	the set of edges at *t*-th snapshot
F	the mapping function for node types
φ	the mapping function for edge types
T	the set of node types
R	the set of edge types
*T*	number of snapshots in set G
$Z^{t}$	the overall node embedding at *t*-th snapshot
*d*	number of final embedding dimension

**Table 2:** Statistics of relation prediction datasets.

Datasets	Types	#Nodes	#Edges	#Node-Type	#Edge-Type	#Snapshots	Time Span
DBLP	Academic	132,582	275,206	3	3	19	19 years
Aminer	Academic	41,901	68,068	3	3	16	16 years
EComm	Commercial	37,724	91,033	2	4	11	11 days
Math Overflow	Social	24,818	506,550	1	3	11	2350 days

**Table 3:** Parameter configurations of the proposed models for relation prediction.

Parameters	Setting
The trade-off of final node embeddings	λ= 0.8
The embedding dimension	d= 128
The random walk length	l= 100
The number of walks per node	50
The number of sampled neighborhood	25
The number of negative samples	5
The number of filters	64
The training rate	0.001
The dropout rate	0.2

**Table 4:** Performance comparison on four datasets for the task of relation prediction on dynamic heterogeneous networks. The best results are highlighted in bold.

Methods	DBLP		AMiner		EComm		Math Overflow	
	AUROC	AUPRC	AUROC	AUPRC	AUROC	AUPRC	AUROC	AUPRC
DeepWalk [13]	0.743	0.762	0.719	0.741	0.564	0.561	0.707	0.756
node2vec [15]	0.747	0.766	0.724	0.746	0.597	0.594	0.713	0.714
GAT [29]	0.762	0.774	0.757	0.751	0.647	0.642	0.735	0.763
GraphSAGE [28]	0.773	0.801	0.761	0.754	0.595	0.590	0.637	0.660
metapath2vec [36]	0.852	0.855	0.795	0.804	0.603	0.653	0.696	0.749
metapath2vec++ [36]	0.853	0.861	0.798	0.811	0.621	0.686	0.706	0.751
HetGNN [35]	0.871	0.863	0.786	0.793	0.646	0.692	0.721	0.734
dyngraph2vec-RNN [30]	0.651	0.679	0.683	0.677	0.499	0.506	0.523	0.562
dyngraph2vec-AERNN [30]	0.672	0.691	0.685	0.684	0.512	0.509	0.582	0.597
DySAT [33]	0.659	0.701	0.693	0.686	0.508	0.511	0.501	0.538
DHNE [18]	0.757	0.766	0.776	0.779	0.553	0.619	0.678	0.721
DyHATR [19]	0.863	0.869	0.832	0.817	0.693	0.731	0.743	0.778
CDHNE-GRU (proposed)	0.886	0.879	0.851	0.833	0.717	0.745	0.762	0.791
CDHNE-LSTM (proposed)	**0.903**	**0.885**	**0.854**	**0.841**	**0.725**	**0.751**	**0.775**	**0.797**

## Data Availability

The data presented in this study are available on request from the corresponding author.
